# Effect of Alkali and Sulfate on the Hydration Characteristic of Cement-Based Materials Containing Coal Gasification Slag

**DOI:** 10.3390/ma15248868

**Published:** 2022-12-12

**Authors:** Zuzhong Li, Fan Li, Haiwei Xie, Weidong Liu, Rui He, Peiliang Cong, Jinhai Zeng

**Affiliations:** 1 School of Materials Science and Engineering, Chang’an University, Xi’an 710064, China; 2 School of Traffic & Logistics Engineering, Xinjiang Agricultural University, Urumqi 830052, China; 3 Guangxi Key Laboratory of Road Structure and Materials, Guangxi Transportation Science and Technology Co., Ltd., Nanning 530007, China

**Keywords:** coal gasification slag, activators, synergistic effect, pozzolanic activity, hydration

## Abstract

Coal gasification slag is an inevitable by-product of the coal gasification process. This paper explored the feasibility of using activators (calcium hydroxide, sodium hydroxide, calcium sulfate, sodium sulfate) to promote the pozzolanic activity of milled coal gasification coarse slags (MCS), and analyzed the effect of alkali and sulfate activators on the hydration characteristic of cement-based materials containing MCS. Coal gasification slags with ignition lossses more than 15% were removed and the remaining slags were considered as cementitious material after milling. Scanning electron microscopy (SEM), X-ray diffraction (XRD), thermogravimetric analysis (TGA) and hydration heat tests were employed to analyze the hydration mechanism of the samples. Besides, the compressive strength values of cement mortars with MCS and activators were evaluated. The results showed that calcium hydroxide was conductive to the formation of hydration products and its crystallization could contribute to the strength improvement of the sample. Calcium sulfate mainly participated in the hydration process of cement to form ettringite (AFt) phases. Sodium hydroxide could accelerate the dissolution of active mineral phases of MCS, resulting in the pozzolanic activity being enhanced. Moreover, sodium sulfate could not only increase the formation of AFt phases, but also improved the alkalinity in sample to facilitate the production of gels. Among them, a better promotion effect could be obtained from the combined application of calcium hydroxide and sodium sulfate. In addition, the compressive strength values of cement mortars containing MCS tended to increase when activators were used. The sample activated by calcium hydroxide and sodium sulfate exhibited the highest strength, increasing by 18.55% at 28 days compared with the sample without an activator.

## 1. Introduction

It is well known that coal gasification technology has been widely applied in the coal chemical industry, contributing to the clean use of coal by controlling pollutant discharge [1,2]. However, the coal gasification process leaves plenty of slags, accounting for around 20% of the total mass of raw coal, including coarse slag and fine slag [3]. The vast accumulating of coal gasification slags not only occupies valuable land resources, but also releases some sulfur-containing or ammonia-containing gases, which cause severe pollution to the surrounding environment.

In recent years, the resourceful utilization of gasification slags has drawn the attention of more and more researchers. Zhu et al. [4] adopted coal gasification fine slags as soil conditioners to effectively improve the physical and chemical properties of alkali sandy soil. Additionally, the gasification slags could be used as silicon fertilizers for promoting the growth of rice [5]. Furthermore, previous studies explored the structural characteristics and mineral composition of coal gasification slags, indicating that gasification slags contain active SiO_2_ and Al_2_O_3_ mineral phases [6,7]. In view of those, some researchers employed coal gasification slags as the admixtures in cement-based materials due to their potential pozzolanic activity [7,8,9]. The results showed that coal gasification slag with proper content could have a nucleation and pozzolanic effect, which is conducive to accelerating the reaction process, facilitating the formation of the hydration products, and refining the microstructure of cement-based materials [8]. In addition, Wu et al. [10] believed that the residual carbon in the slags would hinder the hydration of cement. Thus, the fine slag was not suitable to be used as an admixture in cement-based materials owing to its high content of unburned carbon [6].

In order to further extend the application of industrial wastes (such as blast-furnace slag, fly ash, and coal gasification slags) in civil engineering materials, it is a feasible method to promote their pozzolanic activity by using various activators. For example, some researchers analyzed the effect of sodium sulfate on the hydration and properties of granulated blast-furnace slag. The results showed that the compressive strength of the activated slag pastes could be enhanced at all curing ages [11,12]. Besides, sodium hydroxide could also be applied to promote the pozzolanic activity of blast-furnace slag [13,14,15,16]. For fly ash (FA), many studies have been carried out to improve its activity. On the one hand, sodium sulfate could increase the alkalinity in fly ash cement paste and accelerate the dissolution of fly ash, thus enhancing the compressive strength of concrete at all curing ages [17,18,19,20]. On the other hand, sodium hydroxide and calcium hydroxide were deemed to be effective in accelerating the pozzolanic reaction of fly ash [15,21,22]. For coal gasification slags, Chen et al. [23] prepared geopolymer composites with coal gasification slag and steel slag and found the reaction of hydration and geopolymerization was accelerated by using alkali activators. Xin et al. [24] prepared backfill materials with coal gasification slag, aeolian sand and cement and employed Na_2_SO_4_ and CaO as activators. It could be concluded that the activator type and dosage had a significant impact on the changes of hydration kinetic and mechanical properties of backfill materials. Consequently, this indicates that the utilization of coal gasification slag can be further expanded by using appropriate activators.

The objective of this work was to explore the effect of various activators on hydration and mechanical strength of cement-based materials containing coal gasification coarse slag. Firstly, coal gasification coarse slags (CS) with relatively low carbon content were selected and then were milled. Secondly, the cement pastes containing milled coarse slags (MCS) were prepared with addition of various activators. The hydration mechanism of the cement pastes was analyzed by scanning electron microscopy (SEM), X-ray diffraction (XRD), thermogravimetric analysis (TGA) and hydration heat tests. Finally, the mechanical strength values of cement mortars containing MCS and various activators were evaluated. This research will be conducive to comprehensively understand the activation mechanism of coal gasification coarse slags by various activators and further extend the application of gasification coarse slags in civil engineering materials.

## 2. Materials and Methods

### 2.1. Materials

#### 2.1.1. Selection and Milling of Coal Gasification Coarse Slags

The coal gasification coarse slags (CS) used in this research were supplied by Shaanxi Changqing Energy & Chemical Co., Ltd., Xi’an, China. The oxide compositions of CS were determined by X-ray fluorescence spectrometer (XRF), and the results are presented in Table 1. It can be seen that the main minerals of CS are silicon oxide, alumina, calcium oxide and ferric oxide.

**Figure 1:**
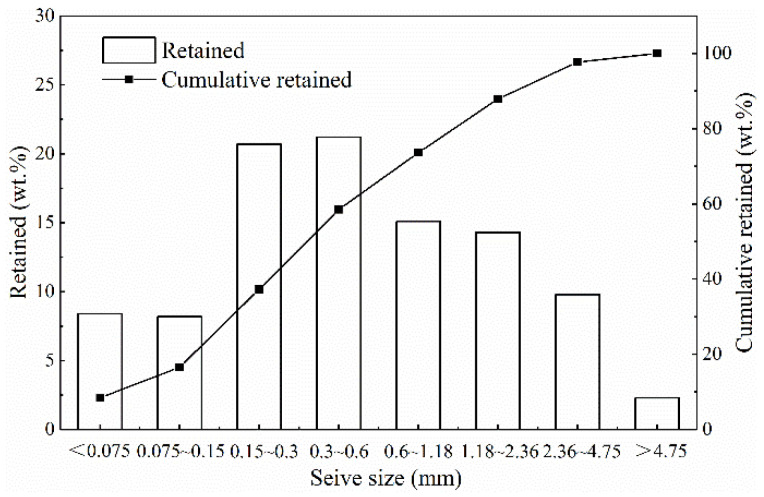
Sieve analysis results of coal gasification coarse slags.

Furthermore, the sieve analysis of CS was carried out according to the aggregate screening method of Chinese standard JTG E42-2005. During the test, the dry coal gasification coarse slags, weighted by 500 g, were screened by hand until the mass of CS on each sieve remained stable. The sieve results are shown in Figure 1. Obviously, the particle size distribution of CS is mainly from 0.15 mm to 2.36 mm.

As was mentioned above, the residual carbon in CS could inhibit the hydration of cement [10]. The ignition loss test is an effective method to distinguish carbon content of CS with different particle sizes. During the test, coal gasification coarse slags weighing about 5 g were placed in a muffle furnace, heated from room temperature to 950 °C and then kept for 15 min. After cooled to room temperature, the samples with different particle sizes were weighed, respectively, and the ignition loss for every sample was calculated. The results are displayed in Table 2.

The ignition loss of CS with particle sizes from 0.15 mm to 2.36 mm is almost more than 15.0%, while that of particle sizes with more than 2.36 mm and less than 0.15 mm are below 7.0%. Generally speaking, the bigger the loss ignition, the higher the content of residual carbon. Thus, it is necessary to screen out CS with high residual carbon content before blending with cement-based materials.

**Figure 2:**
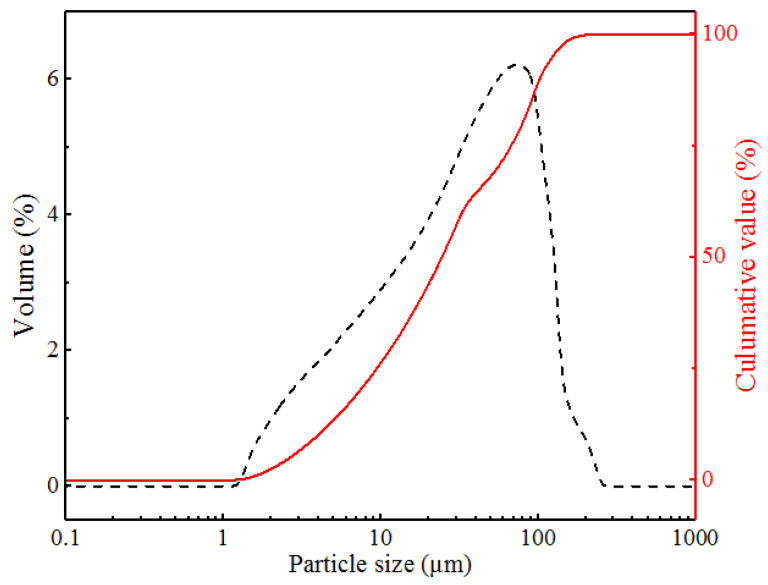
The particle distribution of MCS.

According to the ignition loss results, the CS with particle sizes which were more than 2.36 mm and less than 0.15 showed lower ignition loss, and thus were selected to be admixture of cement-based materials in this study. The selected CS was processed by means of ball milling (SM-500, China), and the milling time was two hours. The particle size distribution of the milled coarse slag (MCS) was determined by using a Mastersizer2000 testing machine, and the results are shown in Figure 2. Moreover, Table 3 shows the physical parameters of MCS.

#### 2.1.2. Activators

The alkaline activators were calcium hydroxide (Ca(OH)_2_) and sodium hydroxide (NaOH), while the sulfate activators were calcium sulfate (CaSO_4_) and sodium sulfate (Na_2_SO_4_). During the experiment process, the soluble activators (NaOH or Na_2_SO_4_) were dissolved in water before experiments and then mixed with cement and MCS. The insoluble activators (Ca(OH)_2_ or CaSO_4_) were mixed with cement and MCS evenly before adding water.

#### 2.1.3. Other Original Materials

Other materials used in tests were listed in Table 4.

### 2.2. Characteristic of MCS

**Figure 3:**
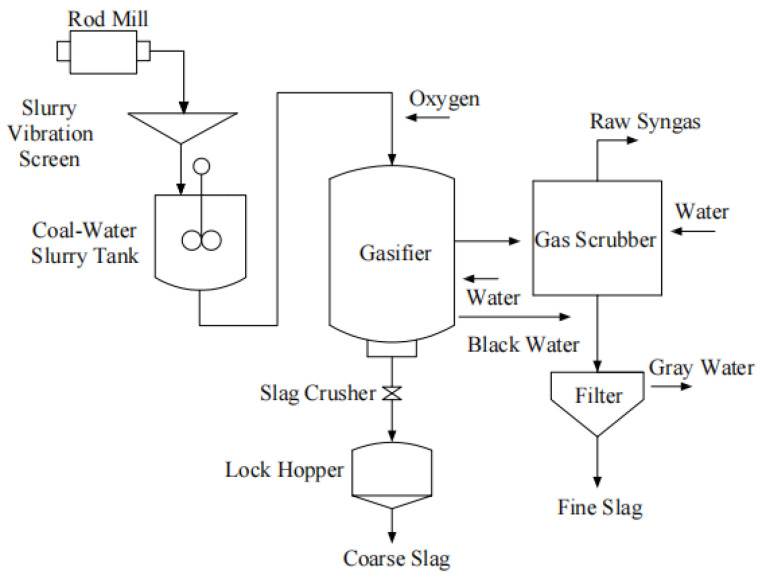
The formation process of CS.

Figure 3 demonstrates the formation process of CS. Typically, CS were collected from the outlet of the lock-hopper. In the process of gasification, molten slag gradually accumulated, then separated from the furnace wall and finally gathered in the bottom of furnace under high-temperature liquid ambiances. The molten slag was chilled after feeding into a slag chamber below the gasifier and then discharged from the gasifier [25].

**Figure 4:**
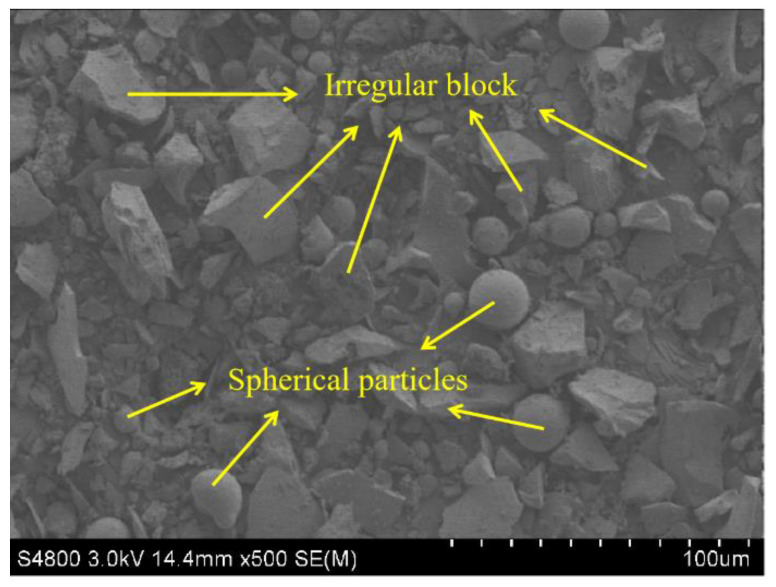
SEM photograph of MCS.

A SEM photograph of MCS is shown in Figure 4. As is demonstrated, most particles of MCS showed irregular block and some small spherical particles could be also observed clearly in the image.

**Figure 5:**
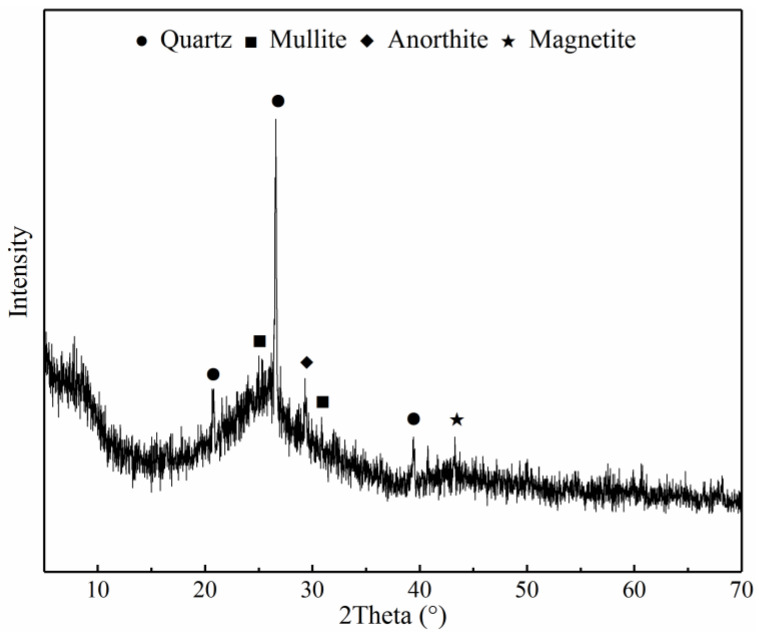
XRD pattern of MCS.

Figure 5 depicts the XRD pattern of the MCS. A small hump in the pattern corresponds to the presence of glassy phase and amorphous material in the MCS, observed at <15° and within 25°–35° of 2θ [26]. It can be found that some mineral phases, such as quartz and mullite, exist in MCS. The majority of those minerals possess pozzolanic activity. Moreover, the activity can be promoted by using various activators.

### 2.3. Characteristic of MCS

#### 2.3.1. Mixture Design

To explore the effect of various activators on the hydration of cement paste (CP) mixed with MCS, the scheme of the cement pastes was designed, including: (a) cement paste mixed with MCS; (b) cement paste mixed with MCS and alkali or sulfate activators; (c) cement paste mixed with MCS, alkali and sulfate activators. All the samples were prepared in the following proportion: cement 0.9, MCS 0.1, water 0.5 (by mass). The selected content of activators (Ca(OH)_2_, NaOH, CaSO_4_ and Na_2_SO_4_) added in the sample were 6%, 1%, 2% and 1% as the mass of binders, respectively. In addition, pure cement paste was cured simultaneously as the control test. When the samples were cured to the specified age, they were soaked in ethanol to stop their hydration, and then dried to constant weight at 60 °C before test.

After that, parallel run phase of the study was to evaluate the mechanical properties of the cement mortar (CM). The ratio of cement paste to sand stands at 1:3 by weight. The mixture ratios of all samples are listed in Table 5.

#### 2.3.2. Sample Preparation

The cement paste samples were placed into the standard curing box (relative humidity was 95 ± 5%, the temperature was 20 ± 1 °C) after mixed evenly, and they were taken out for testing when the curing age reached 7 days. The cement mortar samples were prepared in accordance with the Chinese standard GB/T 17671-2020. The size of the sample used for strength test is 40 × 40 × 160 mm. The preparation process of the sample was divided into two steps. Firstly, the samples were placed in a room-temperature environment for one day before they were demolded. Secondly, the demolded samples were placed in a standard curing box until testing ages were reached.

### 2.4. Analysis Methods

#### 2.4.1. X-ray Diffraction (XRD) Analysis

The hydration products of samples were determined by using a Bruker D8-ADVANCE X-ray diffractometer. All samples were ground into powder that can pass through a 200-mesh sieve before test. The tube current and tube voltage of XRD equipped with a Cu-Ka X-ray source were 40 mA and 40 kV, respectively. The samples were scanned in the region from 5–70° (2θ) at a rate of 10°/min

#### 2.4.2. Thermogravimetric Analysis (TGA)

Thermogravimetric analysis test was conducted by using an SDT 650 thermogravimetric analyzer. The samples were ground into powder when the curing time reached 7 days. During the test, about 5–10 mg samples were placed in an aluminum pan and heated in a nitrogen atmosphere from room temperature to 900 °C at a rate of 20 °C/min.

#### 2.4.3. Scanning Electron Microscopy (SEM)

A small piece of crushed sample was selected and the microstructure of sample was observed by using Hitachi S-4800 scanning electronic microscopy (SEM). The acceleration voltage used in the test was 3–5 kV.

#### 2.4.4. Hydration Heat Tests

The heat of cement paste during hydration process was measured by using a PTS-12S isothermal calorimeter, and the hydration heat of sample was calculated according to Chinese standard GB/T 12959-2008. The temperature of the thermostatic water bath was 20 ± 0.1 °C, and the data collection interval was 5 min. Two parallel tests were carried out for each sample, and the average value was taken when the difference between the two results was less than 12 J/g.

#### 2.4.5. Strength Test

A universal testing machine (CMT-5105) was used to test the compressive strength of mortar samples at 7 and 28 days. The loading rate was 240 N/s according to the Chinese standard GB/T 17671-2020. Each sample contained six test data, with average value reporting.

## 3. Results and Discussions

### 3.1. Analysis of Hydration Products

#### 3.1.1. Hydration Products of Cement Paste

**Figure 6:**
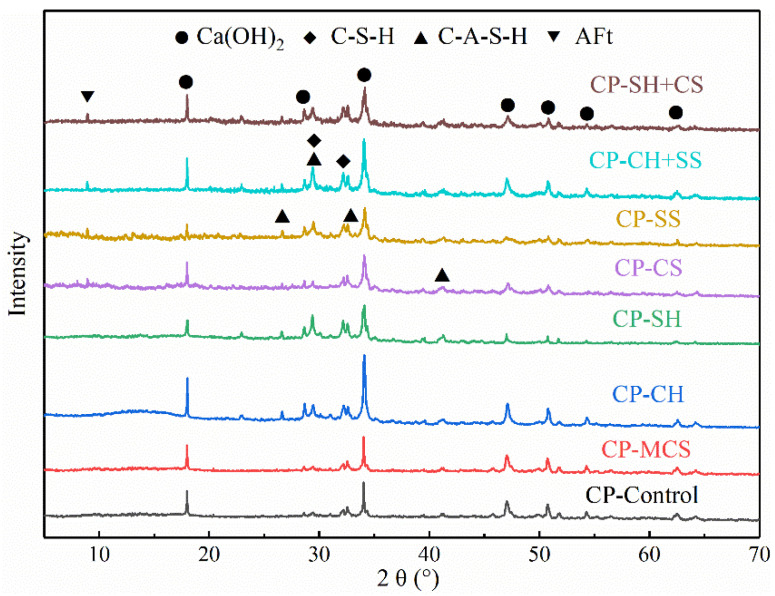
XRD patterns of cement pastes.

The XRD method was adopted to analyze the hydration products of cement pastes which were hydrated for 7 days. The corresponding results are presented in Figure 6. The main hydration products of hardened pastes were gels (C-S-H or C-A-S-H), calcium hydroxide and ettringite (AFt). There was no significant difference for hydration products between CP-MCS and CP-Control, indicating that the pozzolanic activity of MCS is low at the early curing age.

In cement paste, Ca(OH)_2_ produced from cement hydration and alkali activators can both ionize OH^−^ ions, as follows in Equations (1) and (2).

Ca(OH)_2_ → Ca^2+^ + 2OH^−^ (1)

NaOH → Na^+^ + OH^−^ (2)

When MCS contacting with OH^−^ ions, the Si-O-Si and Al-O-Al bonds of active mineral phases can be broken by OH^−^ ions, resulting that silicate and aluminosilicate in MCS were depolymerized and dissolved into the solution [27], as follows in Equations (3)–(5).

Si-O-Si + 3OH^−^ → [SiO(OH)_3_]^−^ (3)

Al-O-Al + 4OH^−^ → [Al(OH)_4_]^−^ (4)

Si-O-Al + 7OH^−^ → [SiO(OH)_3_]^−^ + [Al(OH)_4_]^−^ (5)

After that, the above-mentioned ionic monomers were attracted to each other by hydroxyl groups and formed intermediate complexes, further transforming into oligomeric sols after dehydration and condensation. Finally, the oligomeric sols were interconnected with Ca^2+^ ions and then the hydrated products with three-dimensional network structure appeared in the sample [12], as follows in Equations (6)–(8).

[SiO(OH)_3_]^−^ + Ca^2+^ + H_2_O + OH^−^ → C-S-H (6)

[Al(OH)_4_]^−^ + Ca^2+^ + H_2_O + OH^−^ → C-A-H (7)

[SiO(OH)_3_]^−^ + [Al(OH)_4_]^−^ + Ca^2+^ + H_2_O + OH^−^ → C-A-S-H (8)

Generally speaking, the C-(A)-S-H phases are represented by the peak at 30° (2θ) [28]. With the participation of Al^3+^ ions, C-S-H gel was inclined to turn into C-A-S-H gel [29].

It is obvious that the diffraction peaks of Ca(OH)_2_ and gels in CP-CH were higher than that in CP-MCS. The enhancement of the Ca(OH)_2_ phase was mainly attributed to the addition of Ca(OH)_2_ activator, while the increase of gels phase was caused by the activation effect of Ca(OH)_2_. A previous study confirmed that Ca(OH)_2_ could react with coal gasification slag [6], thus improving the potential pozzolanic activity of MCS.

After the addition of NaOH, the diffraction peak intensity of Ca(OH)_2_ weakened, while that of C-(A)-S-H at 2θ = 29° and 2θ = 33° increased, which means that Ca(OH)_2_ from the cement reaction was partially consumed during hydration and more gels were generated owing to the activation effect of NaOH. Furthermore, the diffraction peak intensity of gels in CP-SH was higher than that in CP-CH; it can be concluded that the activation effect of NaOH is better than that of Ca(OH)_2_ because of its stronger ability to dissolve active minerals.

Generally, SO_4_^2−^ reacts with free Ca^2+^ and [Al(OH)_4_]^−^ in solution to produce ettringite (CaO·Al_2_O_3_·3CaSO_4_·32H_2_O, AFt), as follows in Equation (9):

[Al(OH)_4_]^−^ + Ca^2+^ + OH^−^ + SO_4_^2−^ → AFt (9)

The dissolution rate of aluminosilicate and diffusion rate of ionic monomer were sped up by consuming [Al(OH)_4_]^−^ in sample. The promotion effect of sulfate activators on MCS were reflected in the consumption of [Al(OH)_4_]^−^ to accelerate the dissolution of active minerals, rather than directly dissolve minerals like alkali activators.

As is shown in Figure 6, the diffraction peak of AFt appeared clearly in the pattern with sulfate activators and the diffraction peak intensity of C-(A)-S-H gels was also higher than that in CP-MCS.

In addition, for CP-CS, it is worth noting that CaSO_4_ in paste can react with C_3_A and C-A-H directly, as follows in Equations (10) and (11):

C_3_A + CaSO_4_·2H_2_O + H_2_O → AFt (10)

C-A-H + CaSO_4_·2H_2_O + H_2_O → AFt (11)

CaSO_4_ mainly participated in the hydration of cement, and it had poor solubility. Therefore, calcium sulfate is not an ideal activator for MCS.

Evidently, Na_2_SO_4_ had a significant promotion effect on the pozzolanic activity of MCS from the variation of the diffraction peak intensity in XRD pattern. On the one hand, as follows in Equation (12), Na_2_SO_4_ could react with Ca(OH)_2_ produced by the hydration of cement and produced NaOH, thereby increasing the alkalinity in sample and promoting the dissolution of the active minerals.

Na_2_SO_4_ + Ca(OH)_2_ → CaSO_4_ + NaOH (12)

On the other hand, SO_4_^2−^ from Na_2_SO_4_ can react with [Al(OH)_4_]^−^ to form AFt. Thus, the sodium sulfate in sample played the both roles of alkali and sulfate activators.

As to synergistic activation, the gels diffraction peak intensity of the pastes was higher than that of other samples, showing that the synergistic activation effect of alkali and sulfate on MCS was more obvious than any other single activator. Compared with CP-CH, in the CP-CH+SS, the diffraction peak intensity of Ca(OH)_2_ weakened while that of gels increased obviously. Additionally, the peak of gels in CP-CH+SS was also higher than that in CP-SS. Two reasons can explain such phenomena. On the one hand, Ca(OH)_2_ was consumed by Na_2_SO_4_ and formed NaOH through Equation (12). On the other hand, the increasing of OH^−^ ions content could enhance the alkalinity in sample, leading to easier dissolution of active minerals and production of gels. Hence, the synergistic activation of Ca(OH)_2_ and Na_2_SO_4_ created a better promotion effect on the activity of MCS.

For CP-SH+CS, there were no distinct differences between the pattern of CP-SH+CS and CP-SH, expect for the AFt phase. Such a result further proved that CaSO_4_ mainly participated in the hydration of cement, but did not accelerate the pozzolanic reaction of MCS. In addition, it can be concluded from the above analysis that more C-(A)-S-H gels were generated owing to the effect of NaOH and Na_2_SO_4_.

#### 3.1.2. TGA/DTG Analysis of Cement Paste

**Figure 7:**
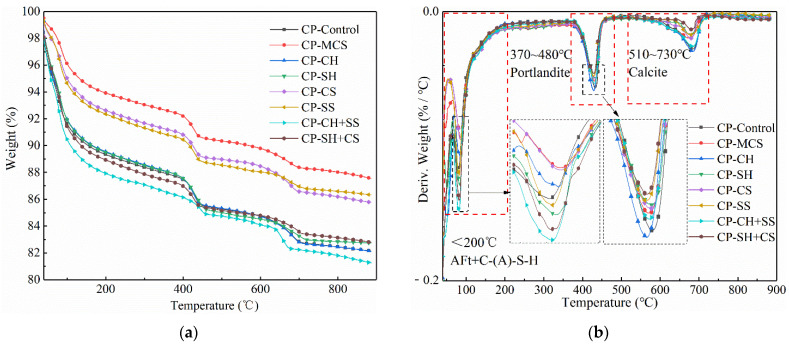
TGA-DTG curves of cement pastes: (**a**) TGA curves of cement pastes, (**b**) DTG curves of cement pastes.

The hydration characteristic of samples was investigated by using thermogravimetric (TG) and derivative thermogravimetric (DTG) analytical techniques. The corresponding results are illustrated in Figure 7. Three distinct weight-loss peaks were observed from room temperature to 900 °C in the DTG curves, as described below [17]:

1. The first weight-loss peak below 200 °C was attributed to the decompositions of C-(A)-S-H gel and AFt as well as evaporation of free water in samples.
2. The second peak between 370 °C and 480 °C was regarded as the decomposition of Ca(OH)_2_ in the samples, which may reflect the activation effect of different activators on pozzolanic activity of MCS. The less calcium hydroxide was left, the deeper the pozzolanic reaction went, and the better the activation effect appeared.
3. The third peak from 510 °C to 730 °C was caused by the decomposition of calcite from the carbonation of calcium hydroxide.

From the results of the DTG curves, compared with CP-MCS, MCS cement pastes with activators exhibited higher weight-loss at the first peak, indicating that more hydration products were formed.

In addition, it could also be observed that the samples with two kinds of activators had the largest weight loss at temperatures up to 200 °C, followed by the samples with a single activator.

For the samples with alkali activators, the intensity of the second peak related to the content of Ca(OH)_2_ increased in CP-CH, and this was mainly caused by the addition of Ca(OH)_2_, while that was observed to decrease in CP-SH, owing to the consumption of Ca(OH)_2_ during pozzolanic reaction. Besides, the sample with NaOH showed higher weight loss below 200 °C, demonstrating that it had a better activation effect than Ca(OH)_2_. In general, the more hydration products, the better the promotion effect. This result was consistent with the previous analysis from XRD.

Based on the DTG data, the first weight-loss peak of CP-CS was close to that of CP-MCS, and this further proved the conclusion that CaSO_4_ was not an ideal activator for mineral admixtures. For CP-SS, it was obvious that more hydration products were generated and Ca(OH)_2_ produced by cement hydration was consumed in the sample. Such a result further demonstrated that the promotion effect of Na_2_SO_4_ was better than that of CaSO_4_. Interestingly, the sample with Na_2_SO_4_ exhibited less weight loss than that with NaOH at the first peak. This demonstrates that although Na_2_SO_4_ can serve as an alkali activator to dissolve active minerals, NaOH has a better direct activation effect. In general, more gels were formed due to the dissolution of active minerals, caused by NaOH and Na_2_SO_4_.

It should be noted that the formation amount of gels and AFt was an important indicator of the activation effect. As illustrated in Figure 7, the addition of alkali and sulfate activators clearly enhanced the activity of MCS due to the relative higher content of hydration products compared with CP-MCS. Furthermore, it can be concluded that the combined application of Ca(OH)_2_ and Na_2_SO_4_ showed a better synergistic activation effect, which was consistent with above analysis.

#### 3.1.3. Microstructure of Cement Paste

**Figure 8:**
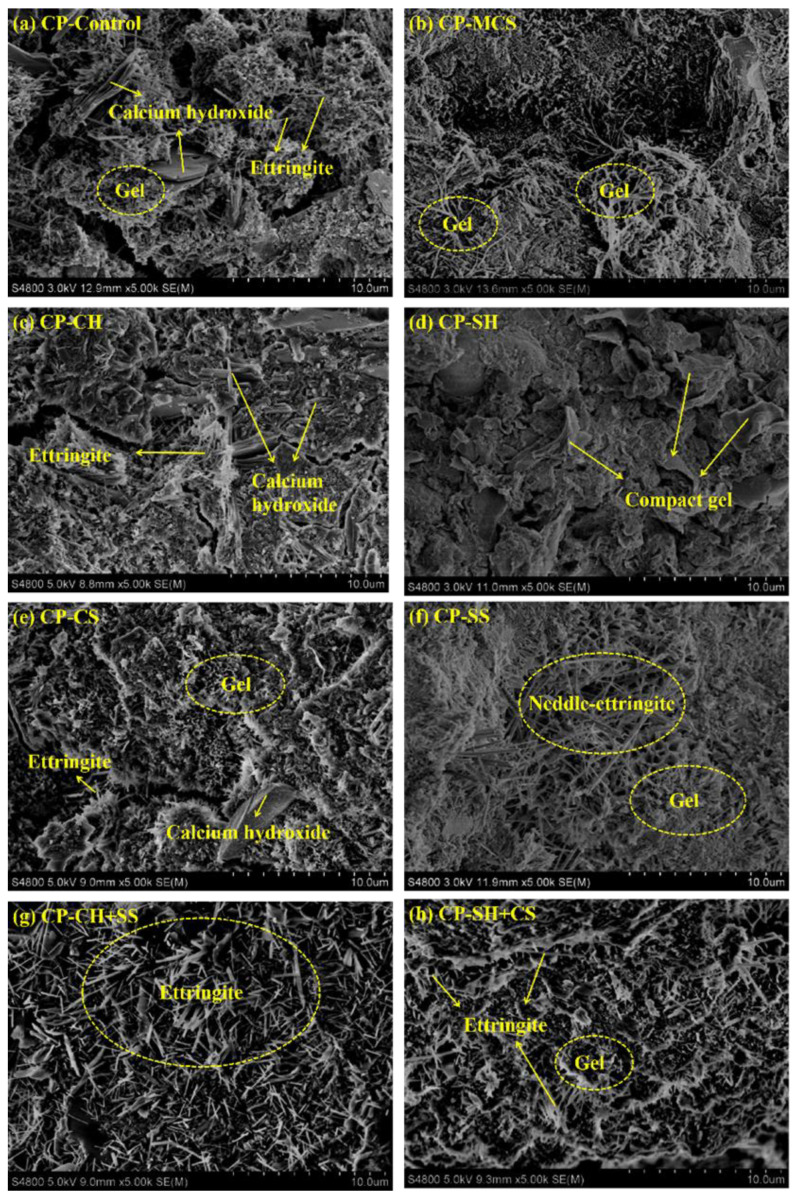
SEM images of cement pastes: (**a**) CP-Control, (**b**) CP-MCS, (**c**) CP-CH, (**d**) CP-SH, (**e**) CP-CS, (**f**) CP-SS, (**g**) CP-CH+SS, (**h**) CP-SH+CS.

Figure 8 shows the microstructure of cement paste samples hydrated for 7 days. From the thorough observation of the Figure, all the samples contained lamellar crystals, amorphous gels and needle-like hydration products, and they were calcium hydroxide, calcium silicate hydrate gel (C-S-H) or calcium aluminum silicate hydrate gel (C-A-S-H), ettringite (AFt), respectively. In pure cement, the calcium silicate hydrate gels presented with a flocculent structure. Compared with CP-Control, CP-MCS showed more interlacing gels covering on the surface of other hydration products, indicating that MCS participated in the hydration process of cement.

As is shown in Figure 8c, CP-CH exhibited a denser structure. Many tabular crystals could be found in the image and lots of hydrated products were attached around them, improving the compactness of hydration products significantly. However, there were not many gels in the image, implying that the addition of Ca(OH)_2_ mainly promoted the crystallization of calcium hydroxide in the sample. Obviously, more compact gels appeared in Figure 8d, which showed that the potential pozzolanic activity of MCS was activated and more gels were generated owing to the addition of NaOH.

As shown in Figure 8e, CP-CS also showed denser microstructure, containing some fine ettringite crystals. As to CP-SS, it can be seen from Figure 8f that lots of interlaced and crowded needle bars were distributed in the sample and amorphous gels could be also clearly observed. It can be concluded that CaSO_4_ was beneficial to the formation of ettringite, filling the void among hydration products, but could not accelerate the reaction between MCS and cement significantly. Nevertheless, for CP-SS, it can be seen that more gels and ettringite appeared in the sample, indicating that sodium sulfate contributed to accelerating the pozzolanic reaction of MCS. Some researchers analyzed the effect of Na_2_SO_4_ on the properties of the CaO activated slags [12]. It was reported that the main hydration products of sample were C-S-H and AFt after the addition of Na_2_SO_4_, which is consistent with the result of this study. Moreover, the addition of Na_2_SO_4_ can fully disperse the raw materials in the sample, resulting in that the reaction between cement and MCS was more sufficient [24].

As can be seen from Figure 8g,h, considerable amounts of hydrated products could be clearly observed in the samples, which demonstrated that the synergistic activation of alkali and sulfate activators caused an obvious effect on the hydration of MCS. Moreover, the sample activated by Ca(OH)_2_ and Na_2_SO_4_ showed a more compact structure with needle-like phases crossing around each other and penetrating evenly in gels. Although CP-SH+CS from Figure 8h contained some fine ettringite crystals and amorphous gels, it showed a relatively loose structure. In conclusion, a better promotion effect on pozzolanic activity of MCS can be obtained from the synergistic activation of Ca(OH)_2_ and Na_2_SO_4_.

#### 3.1.4. Hydration Heat Analysis of Cement Paste

**Figure 9:**
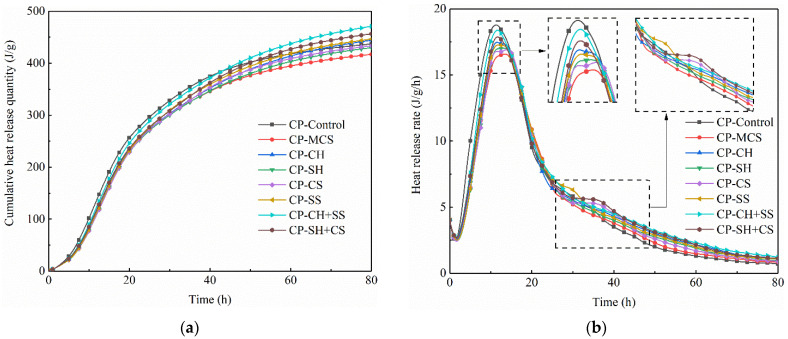
Hydration heat release curves of cement paste: (**a**) cumulative heat release quantity of cement paste, (**b**) heat release rate of cement paste.

The hydration heat test results are presented in Figure 9. It can be seen that the shapes of the heat release rate curves of all samples were similar. The hydration process can be classified into five stages: dissolution, induction, acceleration, deceleration and slow reaction stages, respectively [30].

Compared with CP-Control, the exothermic peak of CP-MCS decreased by 12.09% in the initial period of hydration, owing to the decrease of cement content in the sample and low activity of MCS at early age. It was interesting to note that the heat release rate of CP-MCS was higher than that of CP-Control after about 35 h, which could be attributed to the reaction between MCS and Ca(OH)_2_ produced by cement hydration. Obviously, the addition of activators could advance the arrival of exothermic peak of samples and increase the cumulative heat release quantity, indicating that the pozzolanic reaction was accelerated during hydration process.

For the sample with alkali activators, the heat release rates of CP-CH and CP-SH were greater than that of CP-MCS during the whole experiment, and the initial exothermic peak increased by 5.57% and 3.02%. As mentioned in previous analysis, Ca(OH)_2_ could contribute to the crystallization of calcium hydroxide in the sample, which could shorten the duration of induction stage and make the acceleration stage earlier. Ca(OH)_2_ could also play the role of an alkali activator in accelerating the pozzolanic reaction process, thus increasing the heat release rate and the cumulative heat release quantity. For CP-SH, the exothermic peak did not advance significantly, which indicated that NaOH primarily promoted the pozzolanic activity of MCS and could not affect the induction period of cement hydration.

For the samples with sulfate activator, the exothermic rate curves of CP-CS and CP-MCS were extremely similar, while there was a slight difference in that a small exothermic peak appeared at about 30 h in CP-CS. It should be pointed out that the exothermic peak in CP-CS corresponded to the transformation from AFt to AFm [30]. Such results demonstrated that CaSO_4_ mainly participated in cement hydration and produced more AFt, rather than promoted the pozzolanic activity of MCS significantly. When the activator was Na_2_SO_4_, the initial exothermic peak increased by 4.48% and a small exothermic peak also appeared at about 30 h. Such results showed that Na_2_SO_4_ had an obvious promotion effect on pozzolanic activity of MCS. In addition, it should be noted that in the process of cement hydration, AFt was formed under the condition of sufficient SO_4_^2−^ while AFt was transformed to AFm for lack of SO_4_^2−^ [30]. So, the exothermic peak of transformation from AFt to AFm appeared earlier in CP-SS than that in CP-CS, which may be caused by the low SO_4_^2−^ concentration and result in AFt formation in the sample.

Furthermore, it can be concluded that the samples with both alkali and sulfate activators release more heat than other samples with single activator. The initial exothermic peak of CP-CH+SS and CP-SH+CS increased by 11.2% and 7.98%, respectively, compared with CP-MCS. The more heat release, the more violent the reaction. In conclusion, the synergistic activation with alkali and sulfate was better than any single activator, especially for the combined application of Ca(OH)_2_ and Na_2_SO_4_. Additionally, the reaction between Na_2_SO_4_ and Ca(OH)_2_ could result in the production of heat, contributing to the maximum heat release of CP-CH+SS.

### 3.2. Compressive Strength of Cement Mortar

**Figure 10:**
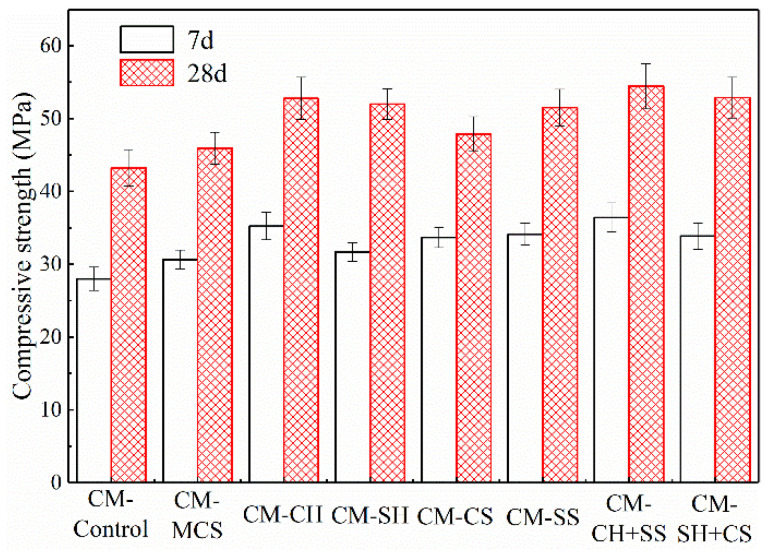
Compressive strength of cement mortar.

The mechanical strength of sample can indirectly reflect the formation of hydration products. The more the products contained, the higher compressive strength shown. The compressive strength values of cement mortars at the curing time of 7 and 28 days are displayed in Figure 10. It is obvious that the strength of samples increased after activators were added. Compared with CM-Control, the compressive strength of CM-MCS increased slightly, which could be ascribed to MCS filling into the pore structure of the hydrated products. Some researchers investigated the microstructure and strength development of quaternary blend high-volume fly ash concrete, and they pointed out that proper content of admixtures mainly acted as a filler at early age, which could play a nucleation effect on hydration process, thereby accelerating the reaction rate and promoting the formation of hydration products [31].

From Figure 10, it can be found that significant improvement on compressive strength was observed after using Ca(OH)_2_, increasing by 14.87% at 28 days. Combined with SEM analysis, a considerable amount of tabular crystals attached with other hydrated products could be observed clearly in CM-CH, indicating that Ca(OH)_2_ could improve the compactness of the structure and contribute to the enhancement of mechanical strength. For CM-SH, based on the previous analysis, more gels were generated owing to the promotion effect of NaOH, being beneficial to the strength improvement of samples. So, the compressive strength of CM-SH was higher than that of CM-MCS, increasing by 13.19%. In addition, although NaOH had a better promotion effect on the pozzolanic reaction, the compressive strength of CM-CH was higher than that of CM-SH, which could be attributed to the fact that the crystallization of Ca(OH)_2_ in sample could improve the compactness of microstructure significantly, thereby enhancing the strength of sample apparently.

The samples with sulfate activators, CM-CS and CM-SS, also exhibited higher compressive strength than the contrast sample (CM-MCS). Adding calcium sulfate and sodium hydroxide increased the compressive strength from 45.93 to 47.88 and 51.52 MPa at 28 days. As was mentioned in the previous analysis, the samples with sulfate activators contained more AFt phases which was favorable for the strength at early curing age. In addition, it should be noted that the incorporation of Na_2_SO_4_ not only participated in the formation process of AFt phases, but also contributed to the production of NaOH which could increase the alkalinity of the sample. That is, the reaction between Na_2_SO_4_ and the Ca(OH)_2_ produced by cement exerted a synergistic effect on the hydration of MCS. Hence, the strength of CM-SS improved significantly.

As to the synergistic activation, the compressive strength of samples with alkali and sulfate activators enhanced apparently. It was proved once again that the combined application of alkali and sulfate activators had a better activation effect on the hydration of samples. When the two samples of CM-CH+SS and CM-SH+CS were compared, the compressive strength of CM-CH+SS increased by 18.55% compared with CM-MCS, while that of CM-SH+CS increased by 15.13%. It can be concluded that better activation effect could be obtained by the incorporation of Ca(OH)_2_ and Na_2_SO_4_.

## 4. Conclusions

In this study, the effect of various activators on hydration characteristic and mechanical strength of cement-based materials containing MCS was investigated. The following conclusions were obtained:

(1) Coal gasification coarse slag contained some active minerals, such as quartz and mullite, which possessed pozzolanic activity. However, the residual carbon in the slags would hinder the hydration of cement. It was necessary to remove the CS with particle sizes which were from 0.15 mm to 2.36 mm. The remaining slags could be considered as cementitious materials after milling.
(2) The pozzolanic activity of MCS could be promoted by incorporating alkali or sulfate activators. Ca(OH)_2_ was beneficial to the formation of hydration products. Its crystallization also improved the strength of sample. CaSO_4_ mainly participated in the hydration of cement to form more AFt phases. NaOH could accelerate the dissolution rate of active mineral phases, resulting in the pozzolanic activity of MCS being enhanced. Na_2_SO_4_ was not only beneficial to the formation of AFt, but also played the role of an alkali activator for increasing the alkalinity in sample. Moreover, the synergistic activation of alkali and sulfate had a more obvious promotion effect on the activity of MCS than any single activator and a better promotion effect could be obtained from the combined application of Ca(OH)_2_ and Na_2_SO_4_.
(3) Adding alkali and sulfate activators could increase the heat release of sample. The sample with sulfate activators exhibited a small exothermic peak at about 30 h, owing to the transformation process of AFt to AFm. Moreover, the heat release rates and the cumulative heat release quantities of the samples with both alkali and sulfate activators were higher than other samples with a single activator, especially for the sample activated by Ca(OH)_2_ and Na_2_SO_4_.
(4) The addition of various activators benefited the strength improvement of samples. Ca(OH)_2_ could improve the compactness of microstructure significantly, contributing to the strength improvement of cement mortar. The formation of gels and AFt could enhance the strength of sample as well. In comparison, the sample with both alkali and sulfate activators together exhibited higher compressive strength, especially the sample with Ca(OH)_2_ and Na_2_SO_4_.

## Figures and Tables

**Table 1:** Chemical compositions of CS (% wt).

Mineral	SiO_2_	Al_2_O_3_	CaO	Fe_2_O_3_	MgO	TiO_2_	Na_2_O	K_2_O	P_2_O_5_	SO_3_
CS	42.59	15.46	12.67	7.78	1.01	1.01	1.37	0.62	0.3	0.27

**Table 2:** Ignition loss of CS with different particle sizes.

Partical Size (mm)	>4.75	2.36–4.75	1.18–2.36	0.6–1.18	0.3–0.6	0.15–0.3	<0.15
Ignition Loss (%)	0.11	0.16	15.87	42.52	64.85	43.32	6.63

**Table 3:** The physical characteristics of MCS.

Indicator	Specific Surface Area (m^2^/kg)	Apparent Density (g/cm^3^)	Loss on Ignition (%)	Water Absorption (%)
MCS	314.18	2.01	1.12	1.06

**Table 4:** List of materials used.

Materials	Type	Specifications
Cement	42.5-type Normal Portland cement	Apparent density: 3.15 g/cm^3^ Specific surface area: 344 m^2^/kg
Sand	River sand	Density in oven-dry condition: 2.60 g/cm^3^ Water absorption: 1.02%
Water	Tap water	

**Table 5:** Mixture ratios of samples (g).

Samples	Cement	MCS	Sand	Water	Activators	Sample Abbreviations
Cement paste (CP)	100	/	/	50	/	CP-Control
	90	10	/	50	/	CP-MCS
	90	10	/	50	Ca(OH)_2_	CP-CH
	90	10	/	50	NaOH	CP-SH
	90	10	/	50	CaSO_4_	CP-CS
	90	10	/	50	Na_2_SO_4_	CP-SS
	90	10	/	50	Ca(OH)_2_ + Na_2_SO_4_	CP-CH+SS
	90	10	/	50	NaOH + CaSO_4_	CP-SH+CS
Cement mortar (CM)	450	/	1350	225	/	CM-Control
	405	45	1350	225	/	CM-MCS
	405	45	1350	225	Ca(OH)_2_	CM-CH
	405	45	1350	225	NaOH	CM-SH
	405	45	1350	225	CaSO4	CM-CS
	405	45	1350	225	Na_2_SO_4_	CM-SS
	405	45	1350	225	Ca(OH)_2_ + Na_2_SO_4_	CM-CH+SS
	405	45	1350	225	NaOH + CaSO_4_	CM-SH+CS

## Data Availability

Not applicable.
